# Universal Polar Instability in Highly Orthorhombic Perovskites

**DOI:** 10.1021/jacs.4c11163

**Published:** 2024-10-16

**Authors:** Cameron A. M. Scott, Nicholas C. Bristowe

**Affiliations:** Centre for Materials Physics, Durham University, South Road, Durham DH1 3LE, U.K.

## Abstract

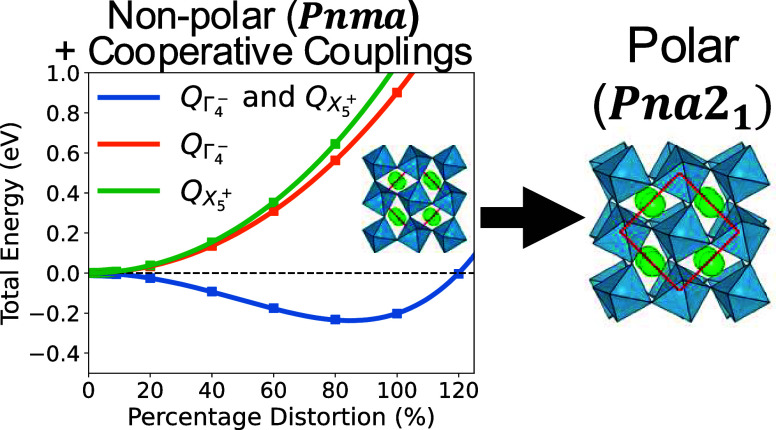

The design of novel multiferroic ABO_3_ perovskites is complicated by the presence of necessary magnetic cations and ubiquitous antiferrodistortive modes, both of which suppress polar distortions. Using first-principles simulations, we observe that the existence of quadlinear and trilinear invariants in the free energy, coupling tilts, and antipolar motions of A and B sites to the polar mode drives an avalanche-like transition to a non-centrosymmetric *Pna*2_1_ symmetry in a wide range of magnetic perovskites with small tolerance factors, overcoming the above restrictions. We find that the *Pna*2_1_ phase is especially favored with tensile epitaxial strain, leading to an unexpected but technologically useful out-of-plane polarization. We use this mechanism to predict various novel multiferroics, displaying interesting magnetoelectric properties with small polarization switching barriers.

## Introduction

Perovskite materials with the ABX_3_ chemical formula provide a fascinating playground for exploring the physics of transition metal compounds. Diverse phenomena including noncollinear magnetism, metal–insulator transitions, (anti)ferroelectricity and superconductivity are known to exists in the structure type. Such a wide range of physical phenomena is enabled by the ability of the structure to distort - largely through tilts and rotations of the B-site octahedra - to accommodate almost any element on the A and B sites. For a given ABX_3_ composition, the tendency toward distortion is phenomenologically described by the tolerance factor,
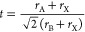
1where *r*_A_, *r*_B_, and *r*_X_ are the ionic radii of the A, B and X sites, respectively. For small variations from the ideal *t* = 1, octahedral distortions occur to alleviate the size mismatch. For very small or very large *t* (typically *t* < 0.7 or *t* > 1.0), distortions can not stabilize the perovskite structure and other structural polymorphs are favored. Such structural and chemical flexibility make perovskites ideal for engineering desired physical properties. However, engineering multiferroism (the simultaneous ordering of both electric and magnetic dipoles which can be reversed by applied external fields) with a strong coupling between the constituent electric and magnetic dipole orders has proved challenging. There are two main reasons for this: (1) polar distortions in perovskites are typically caused by the formation of bonds between B and X sites.^1^ If the B-site possesses the localized *d* electrons necessary for long-range magnetic ordering, such bonds are suppressed by the *d*^0^ criterion^2^ and (2) the octahedral tilts necessary to accommodate a wide range of cations suppress polar distortions.^3,4^

Despite the limitations enforced by the above phenomena, various mechanisms have been identified that result in the coexistence of ferromagnetism and ferroelectricity in a perovskite architecture. For example, lone-pair cations on the A-site separate the magnetic and polar modes onto distinct cations^5,6^ while layered perovskites can break centrosymmetry and allow ferroelectricity in a hybrid improper mechanism.^7,8^ Alternatively, the strong coupling of epitaxial strain to the polar Γ-point phonon^9^ can result in strain-stabilized noncentrosymmetric phases. This distortion is even seen to occur in systems possessing *d* electrons and thus provides a mechanism to circumvent the tendency of *d*^*n*^ systems to form centrosymmetric structures.^10,11^

In this paper, we use first-principles simulations to explore an alternative mechanism to engineer multiferroicity in a wide range of perovskites despite the presence of magnetic cations and large octahedral tilts. Specifically, we observe that couplings of the polar distortion and antipolar motions of B-site cations to various antiferrodistortive modes such as octahedral rotations stabilizes an out-of-plane polarization through an unusual avalanche-related mechanism.^12^ Furthermore, this mechanism appears to be universal to all perovskites with large tilting and does not rely on lone-pairs or *d*^0^ ions (or layering to break centrosymmetry). While not fundamentally reliant on tensile strain, the mechanism is substantially enhanced by its application due to how strain influences the antiferrodistortive modes present in the couplings. Unexpectedly, tensile strain leads to strong out-of-plane polarizations in sharp distinction to previous studies of strain-induced ferroelectricity.^13^ Finally, we show how these principles can be used to create promising multiferroics with strong spin-phonon couplings and illustrate these effects on a few candidate materials.

## Computational Methodology

All simulations are performed using density functional theory (DFT) as implemented in the Vienna Ab initio Software Package (VASP) Version 6.3.2.^14−17^ We use the Perdew–Burke–Ernzerhof exchange correlation functional for solids (PBESol).^18^ We use a high plane wave cutoff energy of 800 eV to ensure convergence for all systems studied as well as a 7 × 5 × 7 Monkhorst–Pack *k*-grid for the  20-atom supercell. Self-consistent field calculations were continued until differences in energies were within a tolerance of 10^–8^ eV. Geometry relaxations were continued until the smallest Hellman-Feynman force was less than 10^–3^ eV/Å. We use projector augmented wave pseudopotentials in all our calculations. A summary of which electrons are treated as valence is presented in Table S4. To better approximate the effect of electron localization and correlation, we use the rotationally invariant formulation of the onsite Hubbard-*U* parameter.^19^ We use a consistent value of *U* = 4 eV for all materials with unpaired *d* electrons and *U* = 0 eV for those without. This is also the methodology employed in previous computational screening studies.^20^ The results are not qualitatively affected by the choice of *U* - see the Supporting Information for details.

To apply epitaxial strain to our systems, we utilize an additional patch to VASP^21^ that fixes selected lattice constants during a relaxation. This simulates epitaxial growth on a substrate for a perovskite film with many layers.

The symmetry analysis was conducted using the INVARIANTS tool of the ISOTROPY Software Suite^22,23^ using a *Pm*3̅*m* parent perovskite cell with the A sites at the corner of the cell.

## Results and Discussion

### Origins of *Pna*2_1_ Instability

**Figure 1:**
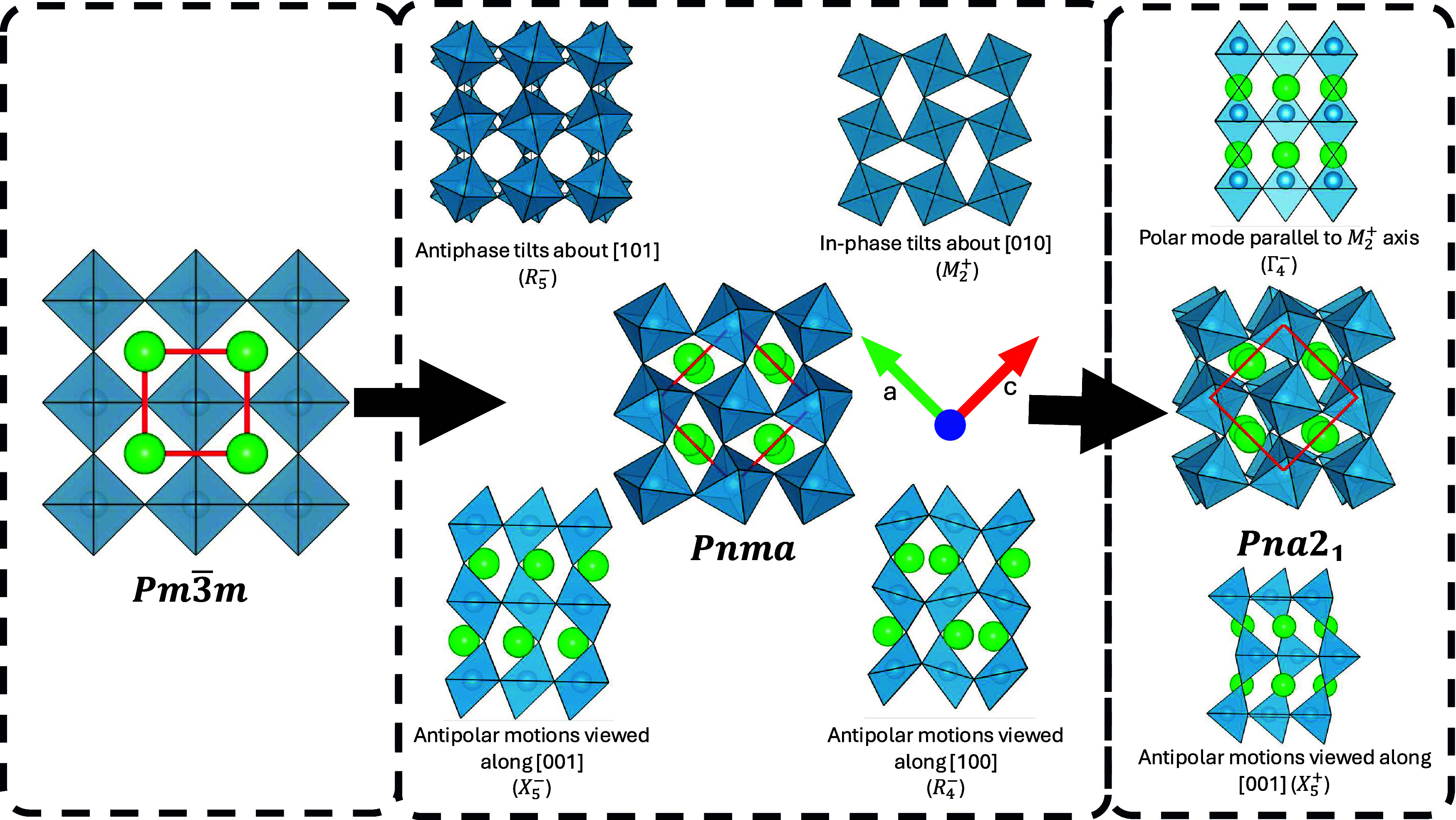
Symmetry adapted distortions leading from the aristotype *Pm*3̅*m* to the orthorhombic *Pbnm* and *Pna*2_1_. The first-phase transition involves the condensation of two separate octahedral rotations and two antipolar distortions resulting in an enlarged unit cell, as denoted by the red square. The second transition introduces the out-of-plane polarization with an additional antipolar motion. Note the distortions are exaggerated for illustrative purposes.

Previous works^24−31^ have demonstrated that perovskites with a low tolerance factor can be stabilized under high-pressure or thin film synthesis techniques and very often form GdFeO_3_ type structures with a *Pnma* symmetry. This phase consists of two primary distortions from the cubic *Pm*3̅*m* reference - equal antiphase tilts about two of the axes of the octahedra and an in phase tilt of a different magnitude about the final axis, a tilt pattern described symbolically in Glazer notation as *a*^–^*b*^+^*a*^–^.^32^ This results in a cell that is  larger than *Pm*3̅*m*. This is depicted in Figure 1 and a summary of the symmetry adapted modes constituting the *Pnma* phase can be found in Table S2.

We explore instabilities of the *Pnma* phase in a wide range of perovskite materials (see Table S1 and caption for details) as a function of epitaxial strain using first-principles simulations (see the Computational Methodology section). We choose to disentangle the effects of lone-pair stereochemistry and magnetically driven symmetry lowering by choosing materials in which the A-site does not possess lone pairs and is also nonmagnetic (*f* electrons were frozen to the core for rare earth cations). To demonstrate the generality of the effect, we also chose to study a selection of chemical compositions covering elements from the *s* – , *p* – , *d* – and *f* – blocks with a range of formal valences and *d* occupancies.

**Figure 2:**
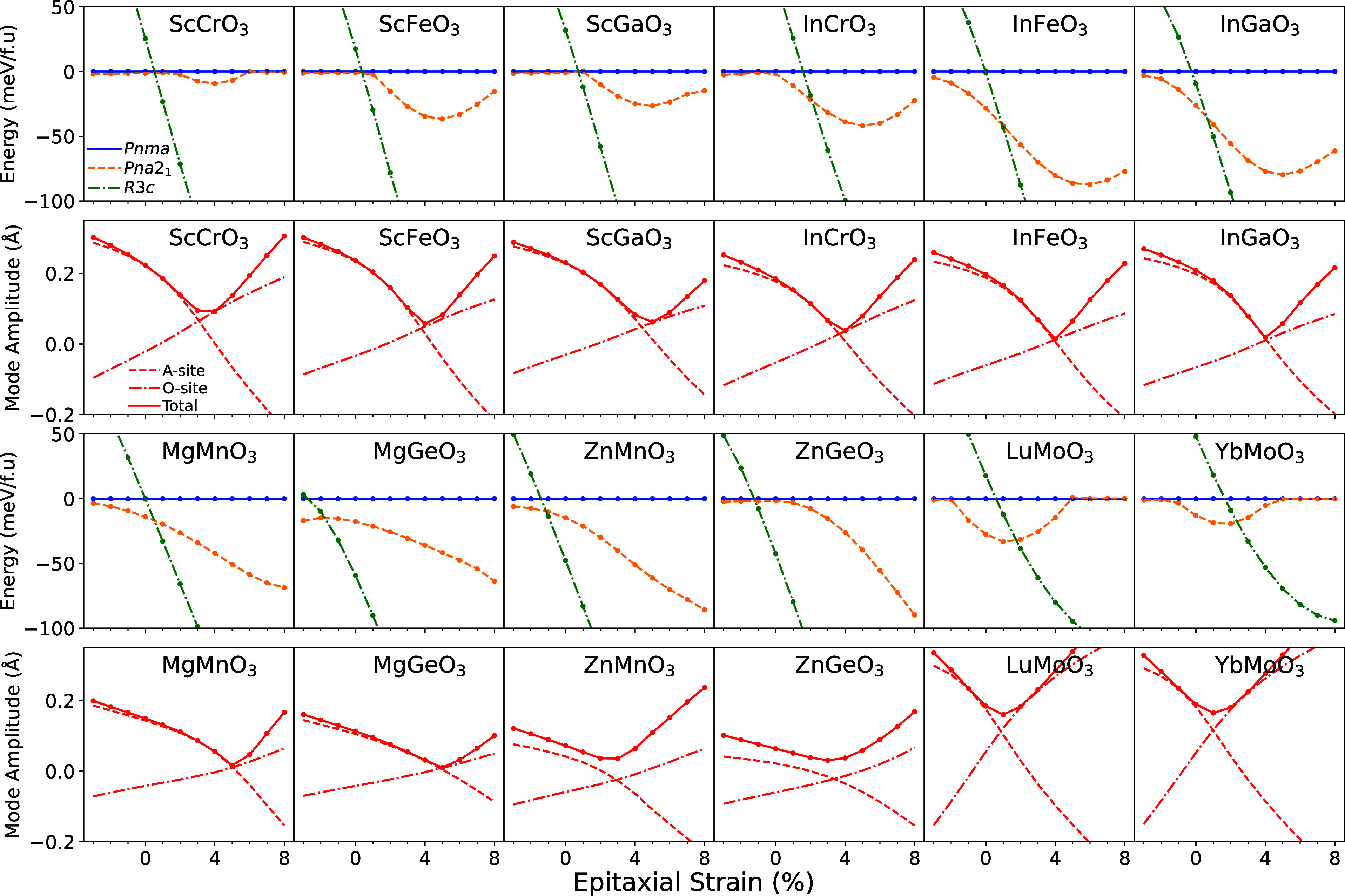
First and third rows: energy of the *Pna*2_1_ (orange dashed) and *R*3*c* (green dash-dot) phases, relative to *Pnma* (blue solid), vs epitaxial strain for a variety of oxide perovskites with small tolerance factors. Second and fourth rows: Total *R*_4_^–^ amplitude as well as the *R*_4_^–^ amplitude decomposed into the components affecting the A and O sites individually. Details of simulations and lattice constants can be found in Table S1.

Despite the large antiferrodistortive modes present in these highly orthorhombic materials, which are known to strongly suppress any polar distortions,^4^ the first and third rows of Figure 2 explicitly show that a prominent instability to a polar *Pna*2_1_ phase is present for many perovskite oxides with low-*t*. This phase is also depicted in Figure 1. The same rows in Figure 2 also demonstrate that the relative phase stability between *Pnma*, *Pna*2_1_ and rhombohedral LiNbO_3_-type *R*3*c* (an extremely distorted phase typically formed when *t* < 0.8 and characterized by *a*^–^*a*^–^*a*^–^ tilts) can be controlled via strain. We do not consider the ilmenite (*R*3̅) structure due to previous reports^33^ indicating that such a structure is higher in energy than the LiNbO_3_-type structure and this is supported in our calculations using InFeO_3_ as a representative example (see Table S3). We initially restrict ourselves to the epitaxial orientation in which the long axis is allowed to relax while the two short axes are fixed to the substrate. This has previously been shown to be the favored orientation within the tensile strain regime that we focus on.^34^ We return to the question of alternate orientations later. Due to the epitaxial constraint forcing the matching of lattice vectors to the substrate, no hexagonal perovskite phases were considered. The *Pmc*2_1_ and *Pmn*2_1_ symmetries with polarization within the plane (there are other distortions available to these phases that are not available to *Pna*2_1_ - see Table S2) were not able to be stabilized under geometry relaxation.^11,34^

All materials studied in Figure 2 show a region of strain in which polar *Pna*2_1_ is stabilized over centrosymmetric *Pnma*. For many materials, this region includes 0%, indicating that strain is not strictly necessary to stabilize *Pna*2_1_. However, there exists an optimum tensile strain at which the energy difference between *Pna*2_1_ and *Pnma* is maximized. Beyond this characteristic optimum strain, the energy difference is reduced until eventually the polarization is destroyed. This can be seen explicity for ScCrO_3_, LuMoO_3_ and YbMoO_3_ and is expected for the rest of the Sc and In series if higher strains were explored. However, the same behavior is not observed in the Mg and Zn series - we explain the diversity of responses to strain later in the section.

In addition, we observe that the LiNbO_3_-type *R*3*c* phase (or *Cc* under biaxial strain) is strongly favored by tensile strain and very rapidly becomes the stable structural polymorph. Nevertheless, the In*B*O_3_ and *R*MoO_3_ families all have a window of stability for the *Pna*2_1_ phase at relatively low strains. Mg(Mn,Ge)O_3_ and ZnMnO_3_ also have a region of stability but exclusively in the compressive regime. Note that we also ran calculations on ZnSnO_3_ (see Figure S1), observing similar trends but an *R*3*c* ground state at all strains (unlike the results of ref (35)).

Two questions are presented by the energy-strain plots in Figure 2; what mechanism is responsible for a universal polar distortion in strongly distorted perovskites and what causes the minima in the energy-strain graph? We begin our discussion by investigating the latter. To do this, we explore how the symmetry-adapted modes of the *Pnma* structure are altered with tensile strain (Table S2 contains a summary of the modes). A representative example is given for InCrO_3_ in Figure S2a. Whereas the antipolar distortion of the *A* sites alone (*X*_5_^–^) and the average of the two tilt modes (*M*_2_^+^ and *R*_5_^–^) remains approximately constant, we observe a pronounced minimum in the small antipolar motion of the *A* and O sites (*R*_4_^–^). Of all the modes, tensile epitaxial strain causes the largest relative change in *R*_4_^–^. In Figure S2b, we show that the *R*_4_^–^ mode can be decomposed into two components that affect only the *A* and O-sites, respectively. Strain causes continuous but opposite changes in both components, with each changing sign, so that there exists a minimum in the total mode amplitude. Figure S2b also shows that this mode minimum neatly overlaps with the minimum of the energy of the *Pna*2_1_ symmetry.

The lowest order coupling terms allowed by symmetry between antiferrodistortive (AFD) modes (such as octahedral tilts and antipolar motions) and polar modes (Γ_4_^–^) have the form
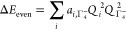
2where *i* iterates over the AFD modes present in *Pnma* and  is the amplitude of the polar distortion. Benedek and Fennie showed that the coupling constants  are large and positive, leading to a suppression of the polar mode.^4^ In particular, their work highlights the small *R*_4_^–^ as having a strong, competitive interaction with the polar mode despite its relatively small size. The materials studied in Figure 2 extend their work to perovskites with even lower *t*, increasing the amplitudes of all AFD modes and subsequently also the importance of the competitive biquadratic interaction. The surprising behavior of the *R*_4_^–^ with tensile strain initially leads to a reduction in this interaction, softening the polar mode just enough to lead to a stable *Pna*2_1_ phase. With additional increase of strain, the *R*_4_^–^ mode then increases leading to the hardening of the polar mode and the eventual loss of metastability for the *Pna*2_1_ structure.

Figure 2 shows identical behavior in all compounds studied; the minimum of *R*_4_^–^ coincides with the stability of the *Pna*2_1_ phase, supporting the importance of the biquadratic coupling between *R*_4_^–^ and the polar mode. The exception is for the Zn and Mg series where the energy does not have a minima in the range of strains studied but the *R*_4_^–^ does. We attribute the lack of an energy minima to the slightly larger tolerance factor in these materials which leads to an overall reduction in all *Pnma* mode magnitudes and a subsequent decrease in all biquadratic interactions.

Having explored how the lowest order even terms in the Landau expansion lead to the presence of the *Pna*2_1_ energy minima, we note that the inclusion of higher order even terms do not add anything substantial to the analysis. Even-order terms that are quadratic in the polar mode and quartic in the tilts have been calculated to have negative coefficients so that extremely large tilts actually favor polarization in certain perovskites with *R*3*c* symmetry.^36^ We show explicitly in Figure S3 that this is not the case for *Pna*2_1_.

**Figure 3:**
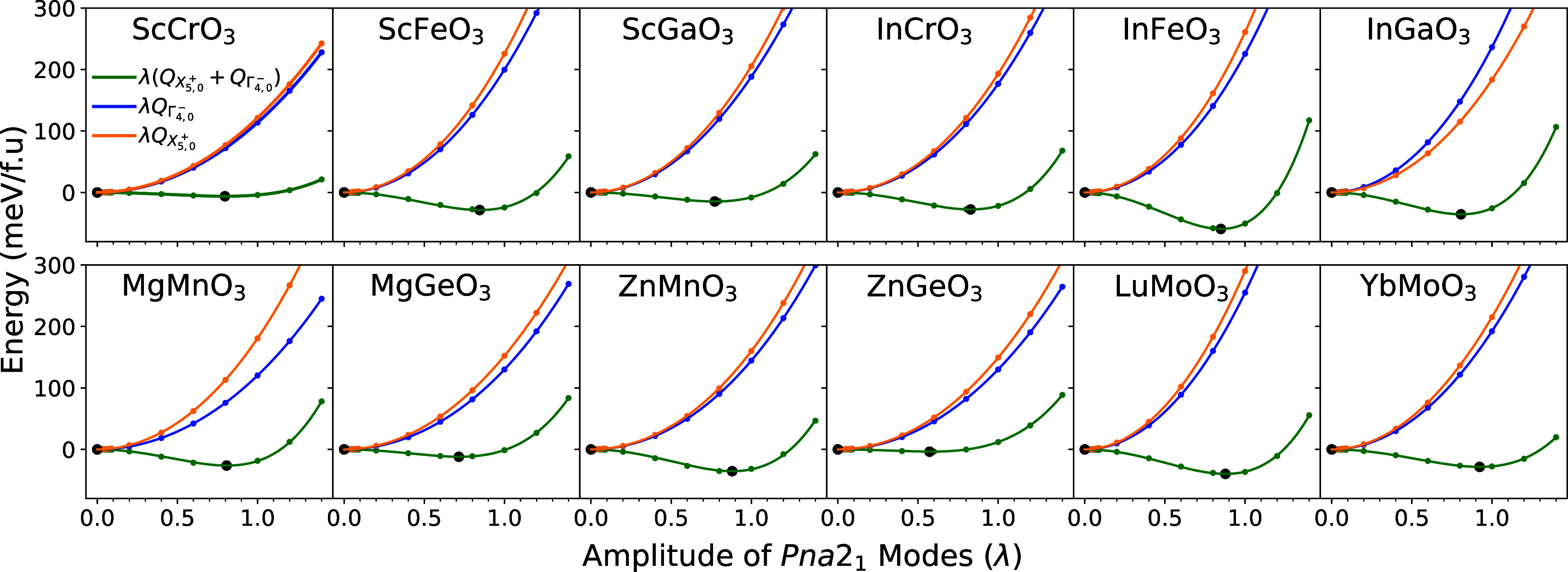
Energy wells formed by scaling combinations of the modes present in *Pna*2_1_ on top of a fully relaxed *Pnma* structure. Modes are scaled via a parameter λ. For example,  = λ, where is the relaxed amplitude of Γ_4_^–^ at 4% strain (2% for rare-earth materials). A double well (minimum indicated by black points) is formed only when both modes are introduced.

To address the former question and understand the origin of the instability, we performed further first-principles simulations in which we took the fully relaxed *Pnma* structure (which already has the *M*_2_^+^ and *R*_5_^–^ tilts as well as the *X*_5_^–^ antipolar mode - see Table S2) at a particular strain (2% for the rare earth compounds and 4% for the others) and investigated how the energy changes as we introduce structural distortions that break the symmetry to *Pna*2_1_. These are the polar mode along the long axis (Γ_4_^–^) and the antipolar motion of cations on the *B* sites (*X*_5_^+^). We have not included the other antipolar *B* site mode (*R*_5_^+^) introduced in the *Pna*2_1_ symmetry because the amplitude in the fully relaxed structures is negligibly small. These results are shown in Figure 3.

Surprisingly, we see that neither the polar Γ_4_^–^ mode nor the antipolar *X*_5_^+^ are unstable when introduced alone. If the two modes are instead introduced together, a double well forms and both modes obtain a nonzero amplitude. It is apparent that both modes must be present in order to produce a polar structure. This idea was further validated by calculating the dynamical force constants of the *Pnma* phase in InFeO_3_ and noticing the existence of an unstable phonon of nearly 50:50 hybrid character. These results also indicate that neither polar Γ_4_^–^ or antipolar *X*_5_^+^ modes are the primary order parameters. Hence, ferroelectricity can not be of proper (unstable Γ_4_^–^) type, and indeed we find nominal Born-effective charges as was also observed in *Pna*2_1_ fluorides.^37^ Nor can ferroelectricity be of the improper (unstable *X*_5_^+^ driving secondary appearance of Γ_4_^–^) type.

We also rule out the possibility that a negative biquadratic coupling constant between *X*_5_^+^ and Γ_4_^–^ is driving the simultaneous appearance of *X*_5_^+^ and Γ_4_^–^. In Figure S4, we show that this constant is actually positive. We therefore conclude that a triggered mechanism^38^ caused by a cooperative biquadratic interaction is not driving the transition to *Pna*2_1_.

To explore other possible origins for the *Pna*2_1_ instability, we enumerated the lowest odd order couplings in the Landau-like expansion. Terms in which both polar Γ_4_^–^ and the antipolar *X*_5_^+^ are coupled at odd order to modes in the *Pnma* structure would explain the unusual behavior of Figure 3, irrespective of the sign of the coefficient. The lowest order terms of this form are
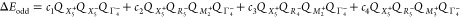
3Some of these couplings were previously identified in a computational study predicting the *Pna*2_1_ symmetry in PbCoO_3_.^39^ The terms above describe how couplings with the antipolar motion of the A-site (*X*_5_^–^), the octahedral tilts (*R*_5_^–^ and *M*_2_^+^) and another antipolar mode affecting both the A-site and O-sites (*R*_4_^–^), all of which have substantial amplitude in small tolerance factor perovskites, can drive the simultaneous appearance of both Γ_4_^–^ and *X*_5_^+^. As we are assuming that the large symmetry adapted modes initially introduced in the *Pnma* symmetry do not switch upon reversal of polarization, both Γ_4_^–^ and *X*_5_^+^ must be switched simultaneously to obtained the degenerate energy state corresponding to reversed polarization. If only one of these modes are reversed, each term in eq 3 acts to increase the energy and no minima (either local or global) is formed. This can be seen for InCrO_3_ in Figure S5.

Behavior of this sort is reminiscent of the avalanche-ferroelectric mechanism studied in Aurivillius compounds.^12,40,41^ Here, the condensation of a single mode forces the condensation of two others. In the case of the present small-*t* perovskites, the large amplitudes of the *Pnma* modes in the above trilinear and quadlinear couplings enable the condensation of both the polar mode and the antipolar *X*_5_^+^ mode. Despite the mathematical similarity, whether these low tolerance factor perovskites should be classified as resulting from an *avalanche transition* is not clear. This classification would require that the transition goes directly from the high symmetry cubic *Pm*3̅*m* to the low-symmetry *Pna*2_1_, While this might occur under certain conditions (e.g., at a particular strain and composition), it seems more likely that the octahedral tilts would condense first to produce *Pnma* and then at a lower temperature, driven by the above couplings, the *Pna*2_1_ phase is stabilized, though this is beyond the scope of this study. This second phase transition could be described as triggered-like since the trilinear (and quadlinear) couplings can renormalize the quadratic terms.^40,42^

To position our work in the context of the existing literature, the *Pna*2_1_ phase is relatively rare in perovskite oxides and there is currently much debate into the causes. The phase has been observed in lone-pair systems like BiInO_3_,^43^ PbRuO_3_^44^ and predicted in PbCoO_3_.^39^ It has similarly been identified in various *d*^0^ materials like CdTiO_3_.^35,45,46^*Pna*2_1_ symmetry has also been observed in rare earth orthoferrites and orthochromates^20,47^ but is usually ascribed to a spin-driven symmetry breaking, although conflicting reports of the *Pna*2_1_ symmetry appearing at a much higher temperature than the rare earth *T*_N_ also exist.^48^ While lone-pair, *d*^0^ and spin-driven effects might be important in certain cases, we argue that the mechanism presented in the present study must also be present and is universal to all low-*t* systems since it depends solely on the symmetry of the parent phase.

Finally, we return to the question of the orientation of the epitaxially *Pnma* phase. The long axis of the *Pnma* phase can either lie parallel to or perpendicular to the substrate surface. We have thus far restricted ourselves to the latter as previous research on CaTiO_3_ has demonstrated that tensile strain tends to favor this orientation.^34^ However, the applicability of this result to our materials is in doubt due to the extremely large distortions. In Table S5, we compute the areas of the face in contact with the substrate for each material (including CaTiO_3_) for both orientations. We see CaTiO_3_ has a larger area with the long axis is perpendicular to the substrate whereas all the small tolerance factor materials - excluding LuMoO_3_ - have larger areas in the orientation where the long axis is parallel to the substrate. Applying a tensile epitaxial strain should favor the orientation that maximizes the area and so it appears that these small tolerance factor perovskites should favor the long axis parallel to the substrate. In Figure S6, we confirm this for InFeO_3_. Interestingly, we see identical physics in the alternate orientation and see a similar polar instability along the long axis - this leads to a polarization parallel to the substrate. However, this new phase is still higher in energy than the alternative *R*3*c* phase and so this structural polymorph is only metastable. At small values of tensile and compressive strains, we still expect to observe a *Pna*2_1_ symmetry with an out-of-plane polarization as this remains the globally stable structure.

We conclude that *Pnma* materials are driven to the *Pna*2_1_ symmetry by odd order terms such as the trilinear and quadlinear terms discussed above. However, these terms favoring *Pna*2_1_ are in contest with the even-order biquadratic terms which inhibit any polarization. Tensile strain manages to reduce the amplitude of the *R*_4_^–^ mode so that its corresponding competitive interaction with the polar modes is lessened and the odd-order terms dominate producing the *Pna*2_1_ symmetry. This promotion of an out-of-plane polarization in the *Pna*2_1_ phase with tensile in-plane strain is opposite to the usual trend found in perovskites which would favor in-plane and disfavor out-of-plane polarization. Further strain starts to increase the magnitude of the *R*_4_^–^ mode leading to the destruction of the polar phase. The crucial contribution of the *R*_4_^–^ mode is particularly pronounced in small tolerance factor perovskites because this mode is larger in these materials, reaching *R*_4_^–^ amplitudes of 0.3 Å or more, considerably larger than the amplitudes of around 0.2 Å or less achieved in the higher *t* Mg and Zn series or those explored in previous studies.^4^ Importantly, this mechanism is only predicted to lead to an orthorhombic cell for relatively small tensile strains -larger strains should result in the rhombohedral *R*3*c* structure, which rapidly becomes the stable polymorph as strain is increased.

### Candidate Multiferroic Systems

**Figure 4:**
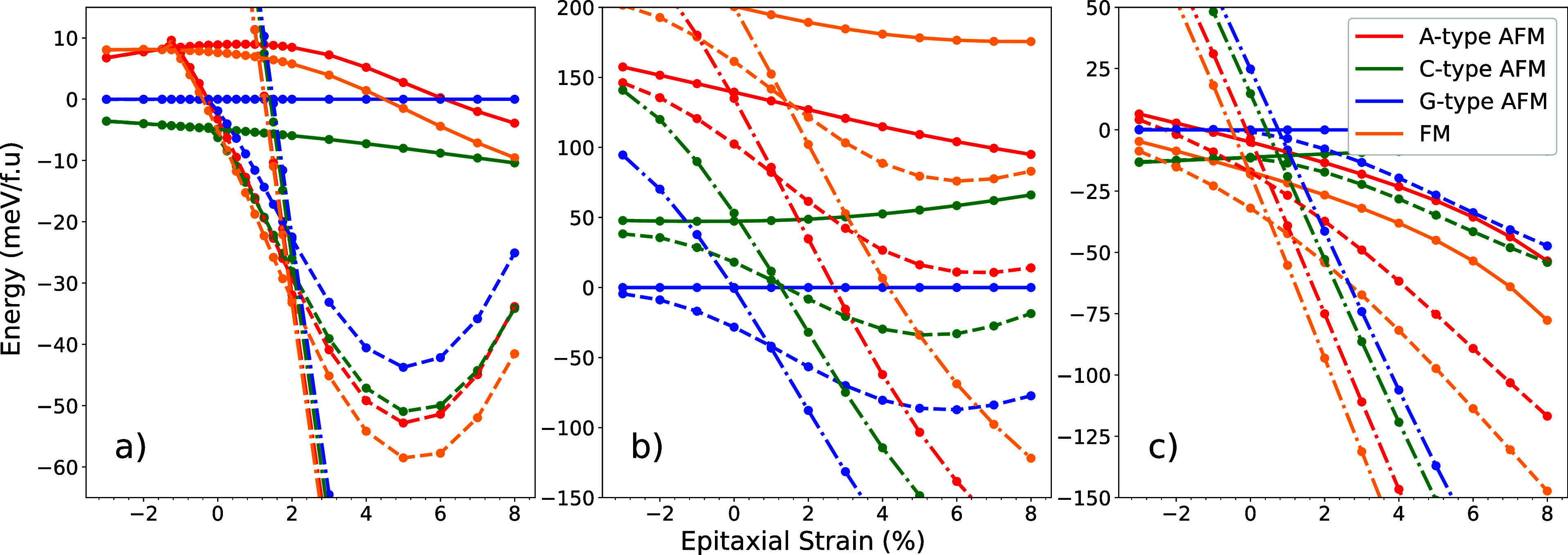
Strain control of magnetic and crystal structure in (a) InCrO_3_, (b) InFeO_3_, and (c) MgMnO_3_. Solid line = *Pnma*. Dashed line = *Pna*2_1_. Dash-dot line = *R*3*c*. Energies with respect to *Pnma G*-type AFM.

The *Pna*2_1_ phase appears to be stable despite several of the materials under investigation being magnetic, allowing for the design of new multiferroics. Figure 4 shows the effect of magnetic ordering on the relative energies of the *Pnma*, *Pna*2_1_ and the *R*3*c* phases as a function of epitaxial strain for three candidate multiferroics: InCrO_3_, InFeO_3_ and MgMnO_3_.

For InCrO_3_, we find the ground state *Pnma* magnetic order to be C-type, in agreement with experiment.^25,26^ Interestingly, the critical strain at which *Pnma* transitions to *Pna*2_1_ changes substantially depending on which magnetic structure is used. For *G* and *C*-type magnetic structures, which have antiferromagnetic spins within each layer of the perovskite cell, the critical strain is approximately 0%. For *A*-type and ferromagnetically aligned spins, the critical strain is instead around −1%. We have seen in the previous section, that the antipolar motion at the B site contributes to the quadlinear and trilinear invariants. The difference in critical strain could be due to a spin-phonon effect in which the ferromagnetic alignment of intralayer spins softens the antipolar B site motion. Due to this spin-phonon coupling, it should be possible to also engineer the *Pna*2_1_ phase posessing a ferromagnetic spin structure, with a compressive strain under an externally applied magnetic field.

Owing to this lower critical strain, ferromagnetism is the magnetic ground state in the region where *Pna*2_1_ is stable. Investigating *Pna*2_1_ at 1% strain, reveals a band gap of *E*_g_ = 1.84 eV - a rare ferromagnetic insulator. At 1% strain, the polarization is 11.6 μC/cm^2^ and the energy difference between the two ferromagnetic orthorhombic structures is Δ*E*_O_ = 29.7 meV/f.u while Δ*E*_R_ = 317.4 meV/f.u between the two rhombohedral (*R*3̅*c* and *R*3*c*) structures. We use these values as a proxy for switching barrier height and predict that the orthorhombic structures have considerably smaller barriers than the rhombohedral materials, providing a potential method to sidestep the high barriers found in *R*3*c* materials.^49,50^

Figure 4b shows the same calculation for InFeO_3_. We do not observe such prominent spin-phonon coupling (possibly due to the presence of *e*_*g*_ orbitals on the Fe^3+^ cation), but the lower barrier present in the orthorhombic structure, presence of high *T*_C_ Fe^3+^, weak ferromagnetic canting and the out-of-plane ferroelectricity make InFeO_3_ a potentially useful thin film multiferroic. MgMnO_3_ (Figure 4c) behaves analogously to InCrO_3_, likely due to the analogous *d*^3^ filling in both, and also displays a ferromagnetic-insulating state over a substantial range of strains. A summary of all calculated material properties is included in Table S3 of the Supporting Information. The Supporting Information also illustrates how our results are altered by the strength of electronic correlation - the existence of a *Pna*2_1_ instability is robust to correlation effects but the strains at which it is achieved are altered.

## Conclusions

Through first-principles calculations and group theoretical analysis, we have explored the key role played by the couplings between AFD modes in the *Pnma* structure to polar distortions in stabilizing the technologically useful *Pna*2_1_ phase with an out-of-plane polarization. Couplings like these lead to an unusual avalanche-like transition in small tolerance factor perovskites. Tensile strains strengthen this effect while also stabilizing the rhombohedral *R*3*c* phase. For small strains, the *Pna*2_1_ phase is predicted to be stable. This mechanism appears resistant to the *d*^0^ rule usually prohibiting the creation of novel multiferroic materials. Using this idea, we identify InFeO_3_ as a potential high *T*_C_ multiferroic with a small polarization switching barrier and sizable wFM moment. We further identify InCrO_3_ and MgMnO_3_ as polar, ferromagnetic insulators exhibiting strong spin-phonon coupling.

## Supporting Information Available

Additional tables and figures describing simulation parameters, additional calculations, and effect of electronic correlation and also an investigation into an alternative epitaxial orientations (PDF)
