# Supplementary material for: Universal Polar Instability in Highly Orthorhombic Perovskites
Source: J Am Chem Soc. 2024 Oct 16;146(43):29735–41. doi: 10.1021/jacs.4c11163 (PMC11528440; doi:10.1021/jacs.4c11163)
Supplement: Supplementary file 1 — ja4c11163_si_001.pdf [file ja4c11163_si_001.pdf]

Universal polar instability in highly orthorhombic perovskites - Supplemental Information

Cameron A.M. Scott and Nicholas C. Bristowe

Centre for Materials Physics, Durham University, South Road, Durham DH1 3LE, United Kingdom

**Material**	t	**Magnetism**	a_{IP} (**Å**)	**Relaxed** $Pna2_1$ X_5^+ (**Å**)	**Relaxed** $Pna2_1$ Γ_4^- (**Å**)
$ScCrO_3$	0.7539	C-type AFM	3.678	0.549	0.854
$ScFeO_3$	0.7426	G-type AFM	3.670	0.814	1.172
$ScGaO_3$	0.7520	NM	3.659	0.706	1.029
$InCrO_3$	0.7737	C-type AFM	3.741	0.728	0.990
$InFeO_3$	0.7620	G-type AFM	3.734	0.899	1.152
$InGaO_3$	0.7717	NM	3.715	0.847	1.121
$MgMnO_3$	0.7786	FM	3.574	0.715	1.164
$MgGeO_3$	0.7786	NM	3.573	0.684	1.147
$ZnMnO_3$	0.7861	FM	3.577	0.815	1.055
$ZnGeO_3$	0.7861	NM	3.568	0.732	0.997
$LuMoO_3$	0.7664	G-type AFM	3.907	0.766	1.087
$YbMoO_3$	0.7688	G-type AFM	3.918	0.657	0.947

Table S1: Parameters of materials studied. Tolerance factor t measured using 6-coordinated A-sites, giving consistency with (Belik, 2014). In plane lattice constant a_{IP} taken as the average bulk $Pnma$ in-plane lattice constants divided by $\sqrt{2}$. Amplitudes of $Pna2_1$ mode, with respect to the $Pnma$ supercell, calculated at 4% strain for non rare-earth materials. For rare-earth materials, the calculation is performed at 2% strain - approximately the minimum of the $Pna2_1$ well. Magnetic structure determined by calculating the energies after bulk relaxation of A-, C-, G- and FM and selecting the lowest energy.

Symmetry Adapted Modes	$Pnma$	$Pna2_1$	$Pmn2_1$	$Pmc2_1$	$R3c$
R_5^-	(a,a,0)	(a,0,a)	(0,a,-a)	(0,a,a)	(a,a,a)
M_2^+	(0;a;0)	(0;0;a)	(a;0;0)	(a;0,0)	N/A
X_5^-	(a,a;0,0;a,a)	(0,0;a;a,0,0)	(0,0;0,0;a,-a)	(0,0;0,0;a,a)	N/A
R_4^-	(a,-a,0)	(a,0,-a)	(0,a,a)	(0,a,-a)	N/A
M_3^+	(0;a;0)	(0;0;a)	(a;0;0)	(a;0,0)	N/A
Γ_4^-	N/A	(a,0,0)	(a,a,0)	(a,a,0)	(a,a,a)
X_5^+	N/A	(0,0;a,-a;0,0)	N/A	N/A	N/A
R_5^+	N/A	(a,0,-a)	(a,0,0)	N/A	N/A
X_2^+	N/A	N/A	(0;0;a)	N/A	N/A
Γ_5^-	N/A	N/A	(0,a,-a)	(0,a,-a)	N/A
M_5^-	N/A	N/A	(a,-a;0,0;0,0)	(a,-a;0,0;0,0)	N/A
X_1^+	N/A	N/A	N/A	(0;0;a)	N/A

Table S2: Symmetry adapted modes of space groups studied. Entries denote the order parameter direction of the individual modes. Order parameter directions should not be confused with real space directions of a distortion. For example, the polar mode Γ_4^- of $Pna2_1$ is along the same real space direction as the M_2^+ rotation axis. In $Pmc2_1$, the polar mode is perpendicular to this tilt axis and in $Pmn2_1$, the polar mode is along the third mutually perpendicular direction. $Pnma$,$Pmc2_1$, $Pmn2_1$ and $Pna2_1$ have the octahedral tilt pattern $a^-c^+a^-$ (or equivalent in the different standard settings) whereas $R3c$ has the $a^-a^-a^-$ pattern. All modes can be visualised using ISODISTORT.

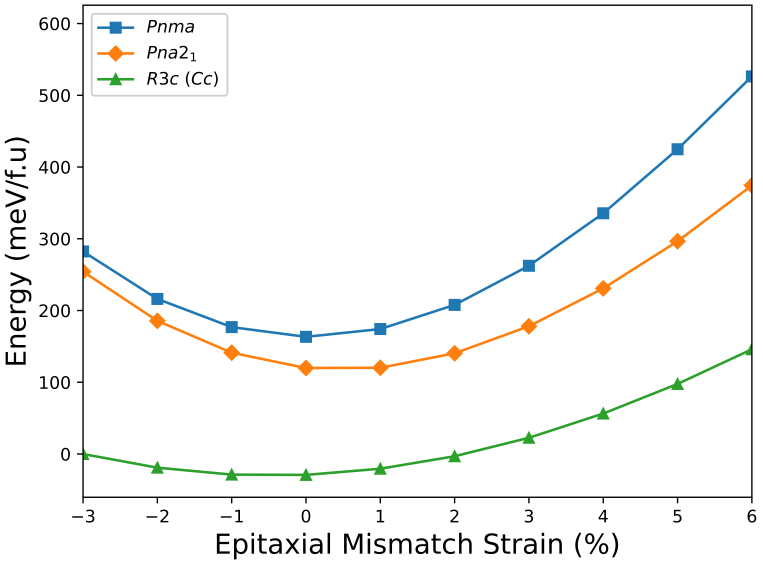

Figure S1: Relative stability, as a function of strain, of $Pnma$, $Pna2_1$ and $R3c$ phases in $ZnSnO_3$. We observe that $R3c$ is the ground state for all values of strain, in contrast to the results of (Kang, 2017).

Material	Strain (%)	Polarization ($\mu C/cm^2$)	Magnetic Structure	Easy Axis	Band Gap (eV)	ΔE_0 (meV/f.u)	ΔE_R (meV/f.u)
$InCrO_3$	1	11.15	FM	[100]	1.84	29.70	317.40
$InFeO_3$	0	22.42	G-AFM*	[001]	1.81	28.30	252.96
$MgMnO_3$	0	15.49	FM	[001]	0.85	13.95	331.01

Table S3: Properties of candidate materials at specified strains. $InFeO_3$ is a G-type antiferromagnetic - the asterisk denotes a magnetic point group allowing for a wFM moment. This has a calculated magnitude of $0.021\mu_B$. ΔE_O and ΔE_R are the difference in energies between the two orthorhombic structures ($Pnma$ and $Pna2_1$) and the two rhombohedral structures ($R\bar{3}c$ and $R3c$) respectively. We use these values as a proxy for ferroelectric switching barrier height. Note that the ilmenite phase is not considered in the current study. Calculations on this phase in $InFeO_3$ reveal the ilmenite structure to be 502 meV/f.u higher in energy than the $Pnma$ phase.

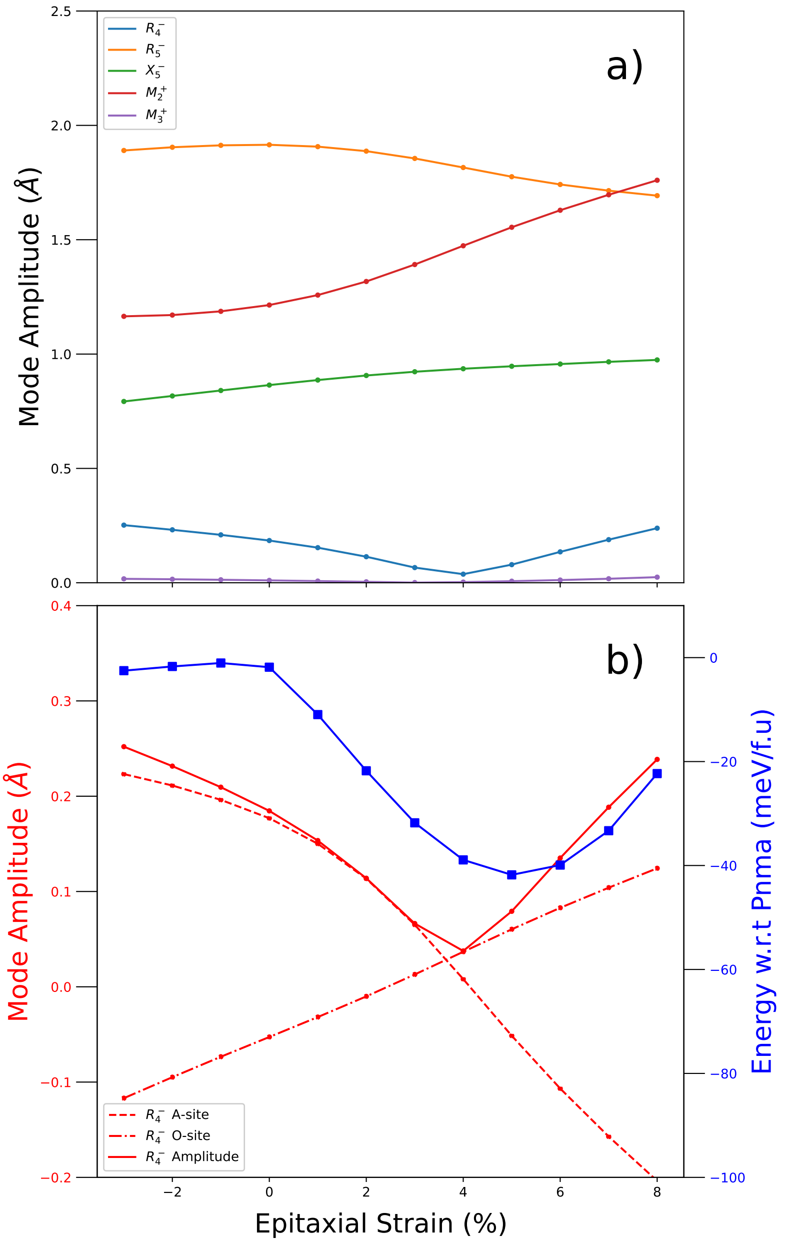

Figure S2: a) Amplitude of modes in $Pnma$ $InCrO_3$. We see a pronounced minimum in the amplitude of the R_4^- mode b) Minimum in R_4^- is caused by opposite trends in its two components. Minimum of R_4^- also coincides closely with the minimum of the $Pna2_1$ energy.

.

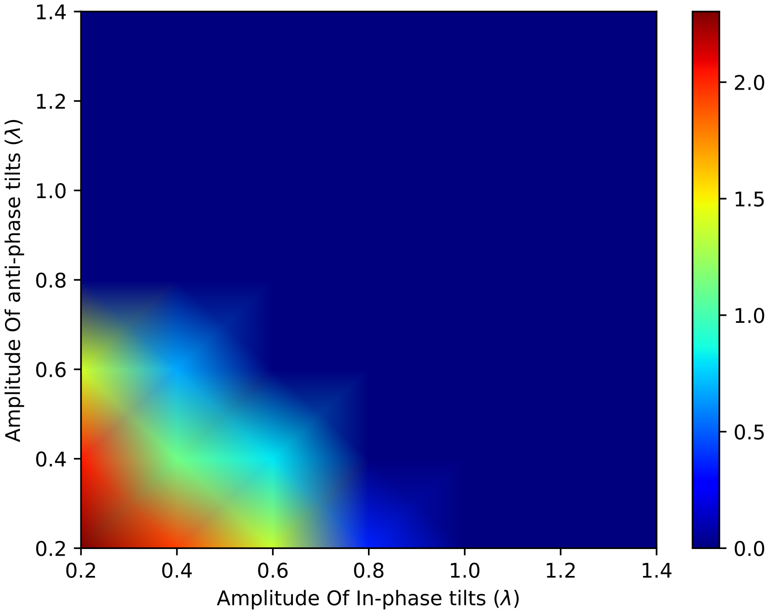

Figure S3: Plot of the amplitude of the polar distortion of 1% epitaxially strained $InFeO_3$. Colour scale denotes the amplitude of the polar distortion, defined as the value of $Q_{\Gamma_{4-}}$ at which the energy is minimised. The deep blue colour for large tilts indicates that the polar mode is not stable when the tilts become large.

**Element**	**Valence Electrons**
Sc	$3s^2 3p^6 4s^2 3d^1$
Cr	$3p^6 4s^1 3d^5$
Fe	$3d^6 4p^2$
Ga	$3d^{10} 4s^2 4p^1$
In	$4d^{10} 5s^2 5p^1$
Mg	$3s^2$
Mn	$3p^6 3d^5 4s^2$
Ge	$3d^{10} 4s^2 4p^2$
Zn	$3d^{10} 4s^2$
Lu*	$5p^6 5d^1 6s^2$
Mo	$4s^2 4p^6 4d^5 5s^1$
Yb*	$5p^6 4f^1 6s^2$
O	$2s^2 2p^4$

Table S4: Valence electrons used in our projector augmented wave ultrasoft-pseudopotential calculations. Asterisks mark rare earth elements for which most or all of the highly localized f electrons have been confined to the core

Material	a	b	c	$A_\perp$	$A_\parallel$
$ScCrO_3$	5.370	7.421	5.033	27.030	27.311
$ScFeO_3$	5.364	7.516	5.018	26.915	27.603
$ScGaO_3$	5.338	7.497	5.011	26.745	27.444
$InCrO_3$	5.387	7.599	5.194	27.977	28.431
$InFeO_3$	5.392	7.692	5.168	27.867	28.726
$InGaO_3$	5.355	7.681	5.152	27.585	28.537
$MgMnO_3$	5.169	7.211	4.940	25.537	25.781
$MgGeO_3$	5.143	7.253	4.963	25.526	25.920
$ZnMnO_3$	5.111	7.295	5.007	25.593	26.100
$ZnGeO_3$	5.078	7.349	5.014	25.464	26.223
$YbMoO_3$	5.721	7.825	5.342	30.565	30.626
$LuMoO_3$	5.719	7.718	5.335	30.510	30.181
$CaTiO_3$	5.442	7.639	5.377	29.266	29.221

Table S5: Fully relaxed *Pnma* materials and their corresponding lattice constants. $A_\perp$ and $A_\parallel$ are the areas of face in contact with the surface and are obtained by $A_\perp = ac$ and $A_\parallel = \frac{b}{2}\sqrt{a^2 + c^2}$.

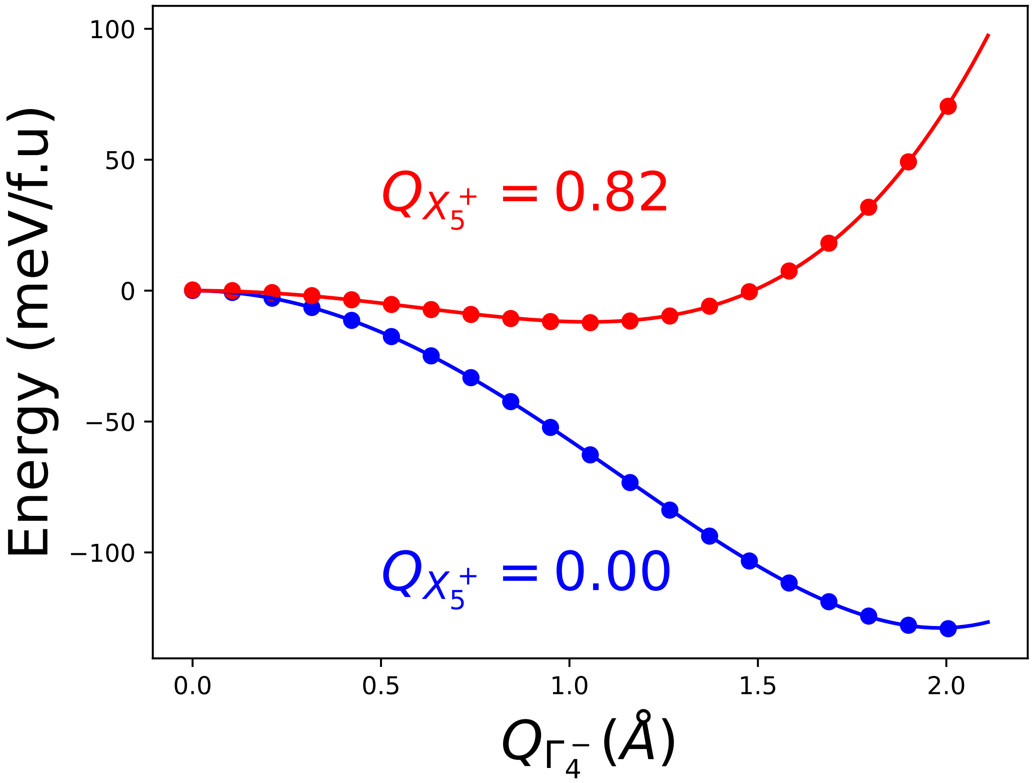

Figure S4: By introducing the polar Γ_4^- mode to the cubic $Pm\bar{3}m$ perovskite cell whilst simultaneously including a fixed magnitude of an antipolar X_5^+ distortion, we demonstrate the positive biquadratic coupling between $Q_{\Gamma_4^-}$ and $Q_{X_5^+}$. A positive biquadratic coupling eliminates the possibility of a triggered-like ferroelectric mechanism between these two modes.

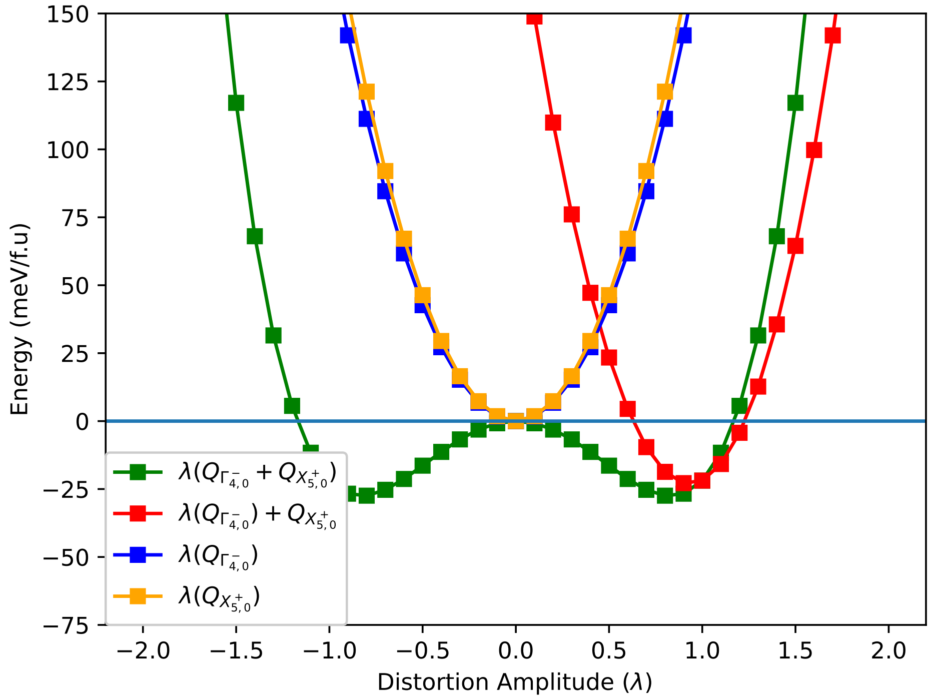

Figure S5: Energy landscape of $Pnma$ $InCrO_3$ as the two distortions leading to $Pna2_1$ are introduced. The green plot shows that landscape obtained by switching both modes simultaneously. A double well is observed indicating that this is the mechanism that switches between two degenerate minimum . The red plot is obtained by switching only the polar mode and keeping the antipolar mode fixed. This produces a single well, suggesting that the polar mode can only be reversed if the antipolar motion of the B-sites is reversed too. The single wells formed by scaling either the polar mode or the antipolar mode are extensions of those shown in Figure 3 and show that the energy is symmetric through the origin.

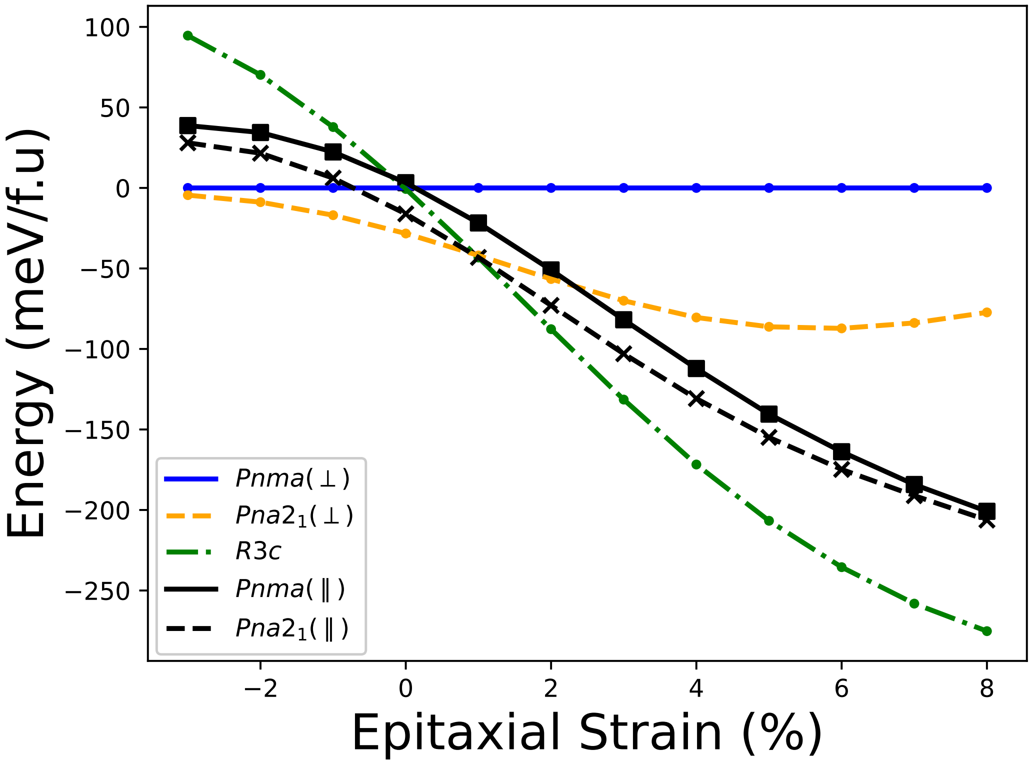

Figure S6: Energy calculations for various phases in $InFeO_3$. We include the three phases from Figure 1 and also the new $Pnma$ phase with the long axis parallel to the plane. We see that this phase is favoured by increasing tensile strain. We also include a phase in which a polar distortion is introduced along the long axis for this new orientation and find that such a distortion is always energetically favourable.

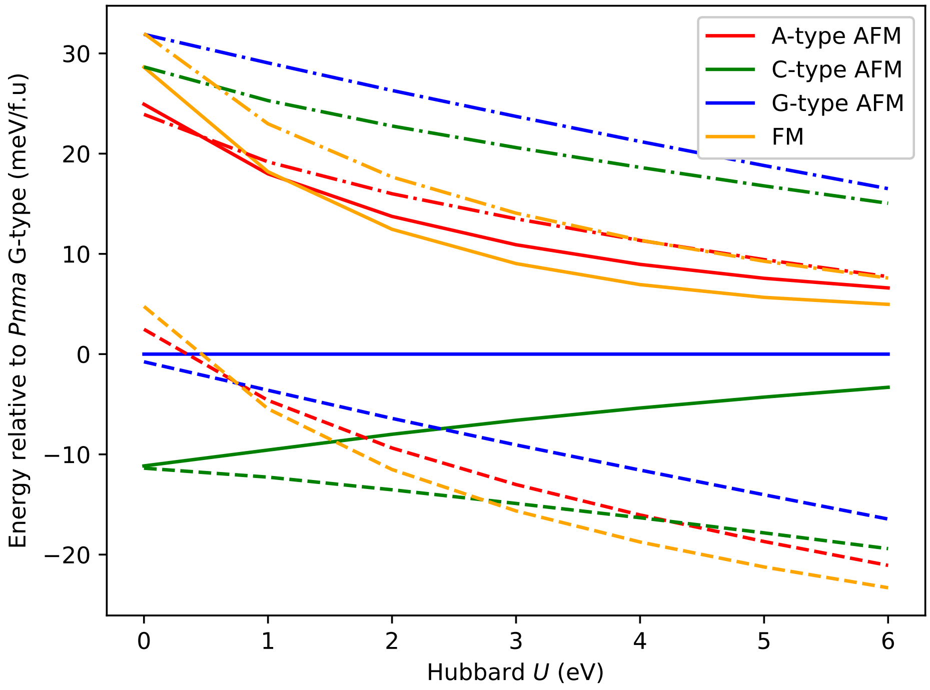

Figure S7: Crystal and magnetic structure of $InCrO_3$ as function of U at 1% strain. Bold lines denote magnetic structure with $Pnma$ crystal symmetry, dashed lines with $Pna2_1$ crystal symmetry and dashdot lines with $R3c$ crystal symmetry. Lowest line denotes lowest energy crystal and magnetic structure for that particular choice of Hubbard-U

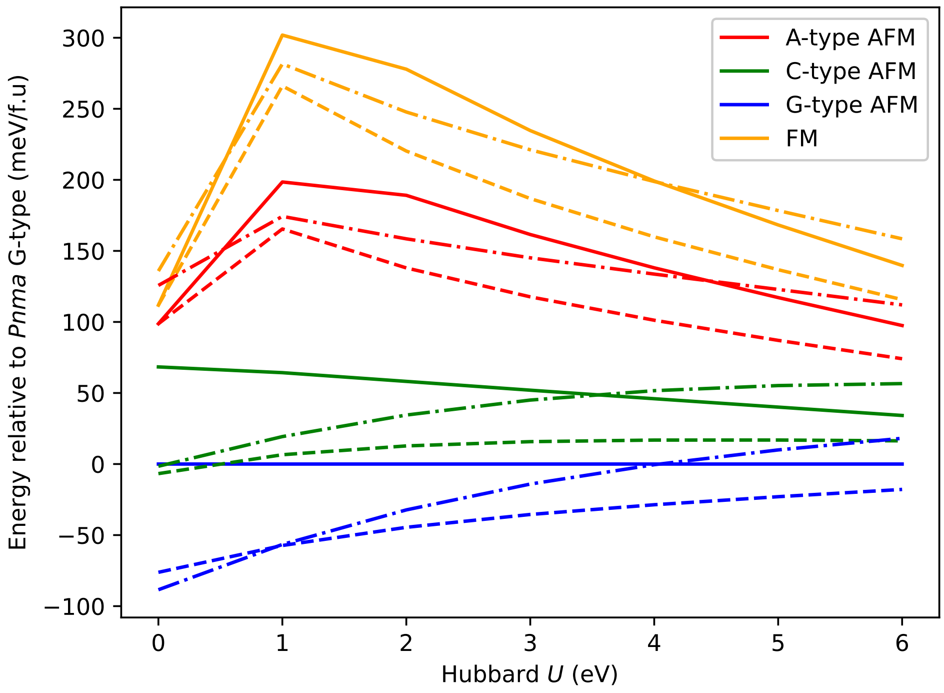

Figure S8: Crystal and magnetic structure of $InFeO_3$ as function of U at 0% strain.Bold lines denote magnetic structure with $Pnma$ crystal symmetry, dashed lines with $Pna2_1$ crystal symmetry and dashdot lines with $R3c$ crystal symmetry. Lowest line denotes lowest energy crystal and magnetic structure for that particular choice of Hubbard-U

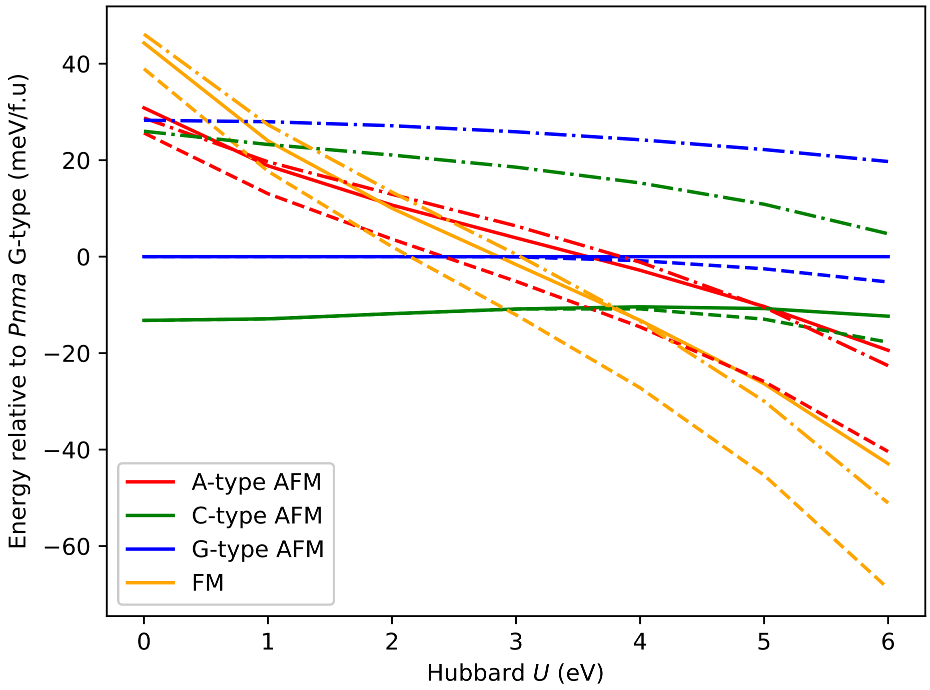

Figure S9: Crystal and magnetic structure of $MgMnO_3$ as function of U at 0% strain.Bold lines denote magnetic structure with $Pnma$ crystal symmetry, dashed lines with $Pna2_1$ crystal symmetry and dashdot lines with $R3c$ crystal symmetry. Lowest line denotes lowest energy crystal and magnetic structure for that particular choice of Hubbard-U
